# Extra Corporeal Membrane Oxygenation (ECMO) in three HIV-positive patients with acute respiratory distress syndrome

**DOI:** 10.1186/1471-2253-14-37

**Published:** 2014-05-21

**Authors:** Francesco Giuseppe De Rosa, Vito Fanelli, Silvia Corcione, Rosario Urbino, Chiara Bonetto, Davide Ricci, Mauro Rinaldi, Giovanni Di Perri, Stefano Bonora, Marco V Ranieri

**Affiliations:** 1 Department of Medical Sciences, University of Turin, Infectious Diseases at Amedeo di Savoia Hospital, Corso Svizzera 164, 10149 Turin, Italy; 2 Department of Surgical Sciences, University of Turin, City of Health and Science, Molinette Hospital, C.so Dogliotti 14, 10126 Turin, Italy; 3 Department of Surgical Sciences, Cardiosurgery Unit, University of Turin, City of Health and Science, Molinette Hospital, C.so Dogliotti 14, 10126 Turin, Italy

**Keywords:** ECMO, HIV, AIDS, HAART, ARDS, Pneumonia, PJP, *Legionella*, Immunocompromised patients

## Abstract

**Background:**

Extracorporeal membrane oxygenation (ECMO) is a life-saving bridging procedure in patients with severe acute respiratory distress syndrome (ARDS). Official indications for ECMO are unclear for immunocompromised and HIV-positive patients affected by severe hypoxemia. Uncertainties are related to prognosis and efficacy of treatment of the underlying disease. However, the care of patients with HIV infection has advanced since the introduction of highly active antiretroviral therapy (HAART), with increased life expectancy and decreased mortality.

**Case presentation:**

Three HIV-infected patients with AIDS were admitted to ICU and were treated with ECMO: a 21 years old Caucasian female with congenital HIV infection presented with *Pneumocystis jirovecii* pneumonia (PJP); a 38 years old Caucasian female with HIV-HCV infection and *L. pneumophila* pneumonia; a 24 years old Caucasian male with fever, cough weight loss and PJP pneumonia. Two patients were alive, with a good immunovirological profile and they went back to their previous quality of life. The last patient died with septic shock after three months of ICU stay.

**Conclusion:**

ECMO was effective in three HIV-positive patients with an otherwise fatal respiratory failure. All patients had severe immunosuppression and/or limited antiretroviral options. A multidisciplinary critical team is needed to individualize the use of ECMO in immunocompromised patients, including those with HIV infection.

## Background

Extracorporeal membrane oxygenation (ECMO) is a feasible life-saving support therapy for patients with severe acute respiratory distress syndrome (ARDS) that is refractory to conventional mechanical ventilation (MV) [1,2]. Key factors for the indication of ECMO are represented by prognosis of the underlying disease, timing, quality of life of survivors and the possibility of being lung transplant candidate [2].

Occurrence of ARDS in immunocompromised HIV-positive in HIV-positive patients is associated to an extremely high mortality especially before the introduction of highly active antiretroviral treatment (HAART) [3]. Under these circumstances, ECMO is usually not recommended in immunocompromised HIV-positive patients that develop ARDS [2,3]. However, nowadays, HIV-positive patients have variable levels of immune function (T-CD4+ lymphocytes/microL) or viral suppression (levels of plasma HIV-RNA) and ARDS may be due to opportunistic infections in antiretroviral-naïve patients or to a bacterial pneumonia during HAART, with considerable differences on the definition of immunocompromised status [3].

We report three cases of successful ECMO support in patients with HIV infection and pneumonia by *Pneumocystis jirovecii* (two cases) and *Legionella pneumophila*.

## Cases presentation

The first patient (female, 21 years old, BMI 15.6 Kg/m^2^, PBW 52.4 kg) had a congenital HIV infection and presented with *Pneumocystis jirovecii* pneumonia (PJP) after a 2-year HAART discontinuation (Table 1). This patient was admitted to the emergency ward with fever, cough, dyspnea and weight loss. Chest CT scan showed diffuse and bilateral ground glass opacities. *P. jirovecii* (PJP) was identified by immunofluorescence (IF), performed on bronchoalveolar lavage (BAL) and other virus, bacterial and fungal infections were excluded with appropriate microscopic, cultural or molecular methods on BAL. Galactomannan test was performed on BAL and blood samples. Two days after the beginning of treatment with steroids and cotrimoxazole, the patient developed mild ARDS [4] that was initially treated with non-invasive mechanical ventilation (NIV) in the Intensive Care Unit (ICU). Later, ARDS evolved to the severe stage [4] and a significant pneumo-mediastinum with subcutaneous emphysema occurred. For this reason, eight days after the onset of respiratory failure, the patient was referred to the regional ECMO center.

**Table 1:** Main clinical characteristics of patients

	**Patient 1**	**Patient 2**	**Patient 3**
	**Perinatally acquired HIV infection**	**Previous IDU with HIV-HCV infection**	**Newly diagnosed HIV infection**
Diagnosis at admission	PJP pneumonia after HAART discontinuation	*Legionella pneumophila* pneumonia	PJP pneumonia
HAART	On HAART for 21 years, multiple changes; low compliance	Compliant, multiple changes	NA
Last HAART	Darunavir/ritonavir, etravirine, enfuvirtide and raltegravir: spontaneously stopped in 2010.	Tenofovir, lamivudine, darunavir/ritonavir	Started on 18 August: Emtricitabine/tenofovir, darunavir/ritonavir, raltegravir, maraviroc
HIV-RNA & CD4/mm3 at admission	(HIV-RNA 386 cp/ml; T-CD4+ 196 cells/mm^3^), HIV-RNA: 118.330 cp/ml; CD4: 2/mm^3^	(HIV-RNA suppressed; T-CD4+ 200 cells/mm^3^), HIV-RNA: 500 cp/ml; CD4: 170/mm^3^	HIV-RNA: 50728 cp/ml; CD4: 3/mm^3^
ICU length of stay (days)	35	22	81
ECMO duration (days)	20	13	24
Antibiotic and antifungal treatment during ECMO	Cotrimoxazole 5 mg/kg q8h*; fluconazole 200 mg; meropenem 1gr q8h.	Cotrimoxazole 5 mg/kg q8h*; levofloxacin 500 mg q12h; rifampin 600 mg/die; pip/tazobactam 4,5 gr q6h; fluconazole 400 mg/die.	Cotrimoxazole 5 mg/kg q8h*; vancomycin 1gr q24h;metronidazole 250 mg q8h.
	On day 2 after ECMO changed to: vancomycin 750 gr *bid,* levofloxacin 750 mg/die, piperacillin/tazobactam 4,5 mg q6h, caspofungin 50 mg/die (with loading dose of 70 mg/die), clindamycin 600 mg q6h and primaquine *per os* ganciclovir 250 mg *bid*, due to concern for reduced plasma concentrations of cotrimoxazole		On day 10 after ECMO changed to: clindamycin 600 mg q6h; caspofungin 50 mg/die (with loading dose of 70 mg/die); primaquine *per os;* colistin 9 MU followed by 4.5 MU *bid*; meropenem 2gr *tid*; liposomal amphotericin B 150 mg; atovaquone 750 mg q6h
	After cotrimoxazole switch to clyndamicin, primaquine and caspofungin there was a slow improvement of respiratory function and ECMO was removed.		After cotrimoxazole switch to clyndamicin, primaquine and caspofungin there was an improvement of general conditions and ECMO was removed.
HAART	Restarted with tenofovir/emtricitabine, raltegravir, darunavir / ritonavir, enfuvirtide and maraviroc	As above	As above

cp= copies; * dose based on trimethoprim component.

Upon arrival at the referral ICU, the patient was intubated, tension pneumothorax was treated and veno-venous ECMO was initiated (19 and 20 F cannulas were inserted into left and right femoral veins, respectively). The by-pass was established with a blood flow of 3 L/min, corresponding to 60% of cardiac output and sweep gas of 5.5 L/min. This setting allowed a super-protective ventilatory strategy that consisted in a tidal volume of 5 ml/kg and a respiratory rate of 5 breaths/min (Table 2). This strategy offered the advantage to rest the lung minimizing the risk of ventilator-induced lung injury [5]. At the third day of ECMO, the patient was successfully extubated and ventilated with non-invasive ventilation (NIV) by facemask. Details of HAART, antibiotic and antifungal treatments are shown in Table 1. After 20 days, ECMO was stopped when arterial blood gases were satisfactory for at least eight hours with a blood flow of 1.5 L/min and a sweep gas of 0.5 L/min (Table 3). After 10 days, the patient was discharged to a step down ICU and then at home. After 1 year of follow-up the plasma HIV-RNA was negative (<20 copies/ml) and the T-CD4+ cell count was 90 cells/microL.

**Table 2:** Ventilator settings before and after institution of ECMO

	**Before ECMO**				
	**VT (ml/kg PBW) or PIP (cmH**_**2**_**O) or SB**	**RR (breaths/min)**	**Pplat (cmH**_**2**_**O)**	**PEEP (cmH**_**2**_**O)**	**PaO**_**2**_**/FiO**_**2**_
**Patient 1**	VT 6	35	28	10	120
**Patient 2**	VT 6	30	29	16	90
**Patient 3**	VT 6	25	28	16	100
	**During ECMO**				
**Patient 1**					
Day1	PIP 20	5	NA	10	150
Day 3	PSV / NIV 5	26	NA	8	200
Day 7	SB	28	NA	NA	180
**Patient 2**					
Day1	VT 5	5	27	16	120
Day 3	VT 5	5	27	16	140
Day 7	PSV 10	22	NA	10	160
**Patient 3**					
Day1	VT 5	5	26	16	100
Day 3	VT 5	5	26	16	90
Day 7	VT 5	5	26	16	90

**Table 3:** Respiratory and ventilator variables before stopping ECMO

	**Patient 1**	**Patient 2**	**Patient 3**
**Main variables**			
FiO_2_	0.4	0.4	0.4
PIP (cmH2O)	SB	10	20
PEEP (cmH2O)	SB	8	8
PaO_2_/FiO_2_	360	250	145
PaCO_2_ (mmHg)	38	42	44
RR (breaths/min)	22	21	26
**Additional variables**			
C_RS_	NA	33	13

Values were obtained after 6–8 hours of sweep gas 0.5 L/min and blood flow 1.5 L/min with gas flow oxygen concentration 0.21.

**List of Abbreviations**: VT (tidal volume), PIP (Peak inspiratory Pressure), SB (spontaneous breathing), RR (respiratory rate), Pplat (plateau pressure), PEEP (positive end expiratory pressure), PaO_2_/FiO_2_ (arterial pressure/inspired fraction of oxygen), C_RS_ (Compliance of respiratory system).

The second patient was a 38 years old female (BMI 20.3 Kg/m^2^, PBW 52 Kg) that had a HIV-HCV infection treated with different HAART regimens, achieving virological suppression (negative plasma HIV-RNA) with T-CD4+ lymphocytes count of 200 cells/microL. After five days of flu-like respiratory symptoms and fever, the patient developed severe respiratory distress and was admitted to a peripheral hospital. Severe ARDS due to pneumonia caused by *L. pneumophila* (urinary antigens) was diagnosed. After two days, the patient was centralized to the ECMO center since refractory to conventional protective MV.

A veno-venous ECMO (21 and 22 F cannulas were inserted into left and right femoral veins, respectively) was established, with a blood flow of 4 L/min (equal to 70% of cardiac output) and sweep gas of 7 L/min with FiO_2_ of 100% (Table 2). After six days of treatment, compliance of respiratory system (28 ml/cmH_2_O) improved as a consequence of resolution of ARDS. Accordingly, the patient was shifted to assisted breathing and seven days later the patient was disconnected from the by-pass when arterial blood gases were satisfactory for at least eight hours with a blood flow of 1.5 L/min and a sweep gas of 0.5 L/min, and the compliance of the respiratory system was 33 ml/cmH_2_O) (Table 3). Details on HAART and antibiotic treatment are given in Table 1.

Eight days after centralization to the ECMO center, the patient developed signs of liver toxicity with jaundice (total bilirubin 28 mg/dl), ascites and elevated liver enzymes (AST/ALT 185/50 UI/mL) requiring interruption of HAART; only levofloxacin monotherapy was continued. On day 22 the patient was transferred to the medical ward and after 6 months of follow-up, she was alive with a good immunovirological profile.

The third patient was a 24 years old male (BMI 23.5 Kg/m^2^, PBW 61 Kg) that was admitted to a peripheral hospital with fever, cough and weight loss (30 kg in the last three months). Chest CT scan showed diffuse and bilateral ground glass opacities and BAL was positive for *P. jirovecii* (PJP). Diagnosis of HIV infection was made and there were no plasma CD4+ lymphocytes detectable. Four days after the beginning of treatment with steroids and cotrimoxazole for PJP, the patient developed hypoxemic acute respiratory failure requiring invasive mechanical ventilation. Ten days after admission, the patient developed severe ARDS and he was centralized to the regional ECMO center. A veno-venous ECMO (21 and 22 F cannulas were inserted into left and right femoral veins, respectively) was established, with a blood flow of 3.5 L/min and sweep gas of 6 L/min with FiO_2_ of 100% (see Table 2). On the second day of ECMO, HAART was started and was associated with virological response but without immune recovery (after 1 month on HAART: HIV-RNA 600 cp/ml; 3 CD4+ lymphocytes/microL). During the ICU stay, he also had a bloodstream infections by *K. pneumoniae* carbapenemase producing (KPC-Kp). Details on HAART and antibiotic treatment are given in Table 1. Full ECMO support remained unchanged for three weeks and it was discontinued only after 24 days when arterial blood gases were satisfactory for at least eight hours with a blood flow of 1.5 L/min and a sweep gas of 0.5 L/min (Table 3). However, the development of lung fibrosis, typically associated with the late phase of ARDS [6] left a severely impaired compliance of the respiratory system. On day 81 the patient was transferred to the ICU where he was initially admitted. After three months by the initial ICU admission the patient died for septic shock.

## Conclusion

To the best of our knowledge, this is the first report on successful extracorporeal life support in patients with HIV infection. There are clinical and ethical considerations about ECMO support in patients with HIV infection and AIDS.

Evidence supporting ECMO as life saving treatment for adults with respiratory failure are growing [1,2,7] However, ECMO is a costly intervention that may carry the risk of futility and serious side effects such as major bleeding and infection. Under these circumstances, indication and selection criteria need to be better defined, especially for the immune-compromised patients. In our reports, ECMO was used in severe, life threatening respiratory failure with etiological diagnosis and knowledge of the full potential of HAART, especially in the first patient who had discontinued treatment in the previous two years.

Extracorporeal Life Support Organization (ELSO) guidelines suggest ECMO when the risk for mortality is at least 50% (as identified by PaO_2_/FiO_2_ < 150 and or Murray score of 2–3) [8]. For immunocompromised patients, the guidelines do not propose absolute contraindications but suggest to consider the individual risks/benefits balance. Only leukopenia with absolute neutrophil count <500/mm^3^ and recent or expanding central nervous system hemorrhages are considered relative contraindications. In patients with HIV infection, ECMO may erroneously be considered futile because of the patient’s prognosis and of the uncertainties regarding the efficacy of HAART. Our reports show that ECMO is not futile when clinicians consider the immune-virological parameters with the full potential of HAART success.

In the first patient there were critical considerations, mainly represented by the concern for barotrauma after failure of NIV; there were HIV-related considerations such as the long history of HIV infection and HAART along with extensive multiclass drug resistance. However, in the last years the availability of new drugs and new classes of anti-retroviral allowed to achieve a high probability of virological success even in patients harboring multi-resistant virus [9]. Moreover, the individual level of compliance has been showed to be highly variable over a long history of HAART [10]. In our case, although a complex 6-drug based HAART was started, an adequate counseling after the discharge from ICU along with rapid clinical improvement reinforced patient’s adherence, with treatment efficacy at 48-week.

*L. pneumophila* is the second most common cause of ICU admission amongst severe community acquired pneumonia (CAP) and PJP pneumonia is a frequent clinical presentation in antiretroviral-naïve patients [11]. Under these circumstances, the issue of ECMO in the last two patients may be more frequently encountered in daily practice. In particular, our second patient, in whom the ongoing HAART resulted in stable immunovirological parameters, may perhaps suggest the inclusion of ECMO into a treatment algorithm for HIV-positive patients undergoing HAART.

Special considerations are needed for the third patients where the futility of ECMO support was confuted by the excellent virological response to HAART, even if the absence of immune function recovery in the short term may not be easily predicted.

Predefined criteria to wean patients from ECMO are still lacking. In our center, satisfactory blood gases, obtained with sweep gas 0.5 L/min and blood flow of 1.5 L/min (gas flow oxygen concentration 0.21) for 6–8 hours, is the main criteria. Moreover, the improvement of respiratory system compliance (Crs >30) is an additional criteria that we consider. In particular, we did not report the value of compliance of the first patient because she was breathing spontaneously when ECMO was stopped.

In conclusion, ECMO was effective in three HIV-positive patients with an otherwise fatal respiratory failure, even with severe immunosuppression or limited antiretroviral options. Two patients went back to their previous quality of life. Unfortunately, the third patient needed a prolonged ICU stay and did not have any recovery of immune function and died after three months by the hospital admission. This case series suggest that a multidisciplinary team, involving critical care and infectious diseases experts, is mandatory to define whether ECMO is an adequate therapeutic option also in immunocompromised patients, including those with HIV infection and AIDS.

## Consent

The collection of data was approved by the local Ethical Committee regarding the use and collection of patients data within the ECMO support program, Città della Salute e della Scienza di Torino, Molinette S. Giovanni Battista Hospital, Turin, Italy. Written informed consent was obtained from the patients or next of kin for publication of these case reports. A copy of the written consent is available for review by the Editor of this journal.

## Abbreviations

ECMO: Extracorporeal membrane oxygenation; MV: Mechanical ventilation; ARDS: Severe acute respiratory failure; HAART: Highly active antiretroviral treatment; PJP: *Pneumocystis jirovecii* pneumonia; NIV: Non-invasive mechanical ventilation; ICU: Intensive care unit; VILI: Ventilator induced lung injury; CAP: Community acquired pneumonia.

## Competing interests

The authors declare that they have no competing interests and no specific funding was received.

## Authors’ contributions

FDR, VF and VMR contributed to study design, data collection, drafting and writing the manuscript, including revision. SC contributed to data collection and drafting the manuscript. SB, GDP, RU, CB, DR and MR contributed to study design, supervision and critical revision of the manuscript for intellectual content. All authors read and approved the final manuscript. FDR and VF equally contributed to this work.

## Pre-publication history

The pre-publication history for this paper can be accessed here:

http://www.biomedcentral.com/1471-2253/14/37/prepub
